# Drug discovery and formulation development for acute pancreatitis

**DOI:** 10.1080/10717544.2020.1840665

**Published:** 2020-10-29

**Authors:** Xue Jiang, Ya-Wen Zheng, Shihui Bao, Hailin Zhang, Ruijie Chen, Qing Yao, Longfa Kou

**Affiliations:** a Municipal Key Laboratory of Paediatric Pharmacy, Department of Pharmacy, The Second Affiliated Hospital and Yuying Children’s Hospital of Wenzhou Medical University, Wenzhou, China; b School of Pharmaceutical Sciences, Wenzhou Medical University, Wenzhou, China; c Central Laboratory, The First Affiliated Hospital of Wenzhou Medical University, Wenzhou, China; d Department of Children’s Respiration Disease, The Second Affiliated Hospital and Yuying Children’s Hospital of Wenzhou Medical University, Wenzhou, China

**Keywords:** Acute pancreatitis, inflammation, traditional Chinese medicine, drug delivery, nanoparticles

## Abstract

Acute pancreatitis is a sudden inflammation and only last for a short time, but might lead to a life-threatening emergency. Traditional drug therapy is an essential supportive method for acute pancreatitis treatment, yet, failed to achieve satisfactory therapeutic outcomes. To date, it is still challenging to develop therapeutic medicine to redress the intricate microenvironment promptly in the inflamed pancreas, and more importantly, avoid multi-organ failure. The understanding of the acute pancreatitis, including the causes, mechanism, and severity judgment, could help the scientists bring up more effective intervention and treatment strategies. New formulation approaches have been investigated to precisely deliver therapeutics to inflammatory lesions in the pancreas, and some even could directly attenuate the pancreatic damages. In this review, we will briefly introduce the involved pathogenesis and underlying mechanisms of acute pancreatitis, as well as the traditional Chinese medicine and the new drug option. Most of all, we will summarize the drug delivery strategies to reduce inflammation and potentially prevent the further development of pancreatitis, with an emphasis on the bifunctional nanoparticles that act as both drug delivery carriers and therapeutics.

## 1. Introduction

Acute pancreatitis is a violently acute inflammatory disease, whose clinic symptoms are a sudden continuous abdominal pain accompanied by nausea, vomiting, fever and abdominal distension. The pathology of acute pancreatitis was mainly characterized by stromal edema, vacuolar accumulation, acinar cell necrosis, and inflammatory infiltration of macrophages and neutrophils (Pandol et al., 2007). In mild acute pancreatitis (MAP), the pancreas exhibited limited inflammation, and usually, minimal organ dysfunction. However, when the inflammation of pancreas extends further to peripheral organs, it is called severe acute pancreatitis (SAP), which is mainly presented as pancreatic necrosis, systemic inflammatory response syndrome, and severe multiple organ failure (MODS). SAP often appears a variety of complications, such as pancreatic abscess, pancreatic pseudocyst, shock, organ function failure, and secondary infection of the abdominal cavity, respiratory tract, and urinary tract. The infection diffusion could further cause septicemia. As a result, despite the great advances in critical care medicine for decades, in SAP patients, which accounts for about 20% of total acute pancreatitis patients, the mortality rate is approximately 30% (Portelli & Jones, 2017). Thus, it is of clinical importance to improve the current therapeutic strategies against acute pancreatitis.

Acute pancreatitis is a disorder that has numerous causes and obscure pathogenesis, which have been intensively investigated but still have not reach a consensus. Several well-known pathological theories of acute pancreatitis include pancreatic ductal obstruction secondary to gallstones, alcohol abuse, endoscopic retrograde cholangio pancreatography (ERCP) and various drugs. These factors could trigger the pathological cellular pathways and organelle dysfunction that culminate in the acinar cell death, local and systemic inflammation, which leads to irreversible pancreatic tissue damage and dysfunction (Gukovskaya et al., 2016; Lugea et al., 2017). Recently, with the improvement of people's living standards, hyperlipidemic pancreatitis becomes the third disease after alcoholic pancreatitis and biliary pancreatitis. But still, in up to 15% of people with acute pancreatitis, the causes are unknown.

To date, no effective therapeutic agents exist to treat or prevent acute pancreatitis directly. More often, the current drug therapy is used to ease the symptoms, for instance, inhibiting the pancreatic enzyme secretion or mitigating the sharp pain. Once diagnosed with acute pancreatitis, fluid resuscitation, also named nutrition support, is often used to improve intestinal and pancreatic microcirculation perfusion, avoiding the ischemia-induced secondary pancreatic infection and bacterial translocation (O’Brien & Omer, 2019; Ramanathan & Aadam, 2019). Enzyme therapy that aims to restore the balance of pancreatic secretion to ease the pain was also employed in clinic for the treatment of acute pancreatitis. Hence, morbidity and long-term sequelae remain substantially high, reaching about 10% (Yadav et al., 2012; Umapathy et al., 2016). In fact, about 18% of acute pancreatitis patients after clinical treatment experience recurrence, and 8% develop chronic pancreatitis (Vipperla et al., 2014; Ahmed Ali et al., 2016).

The grim situation of acute pancreatitis treatment partly attributes to the lack of effective drug therapy. The current medications usually failed due to the following problems. One is the blood pancreas barrier (BPB), which hinders the efficient drug delivery from the blood supply toward the inflammatory pancreas, and the other one is that single drug might not be sufficient to manage acute pancreatitis owing to a variety of complex pathologies (García-Rayado et al., 2020). Indeed, the complicated causes and pathologies make the clinic treatment of acute pancreatitis extremely difficult. In recent years, substantial studies have revealed that many Chinese medicine active constituents, such as rutin, quercetin, resveratrol, curcumin, and berberine, could effectively increase pancreatic blood flow and significantly improve pancreatic microcirculation through multiple mechanisms (Dong et al., 2019). The application of Chinese medicine formulation for the pancreatitis treatment have reached a consensus, but still lack enough attention.

**Figure 1.**
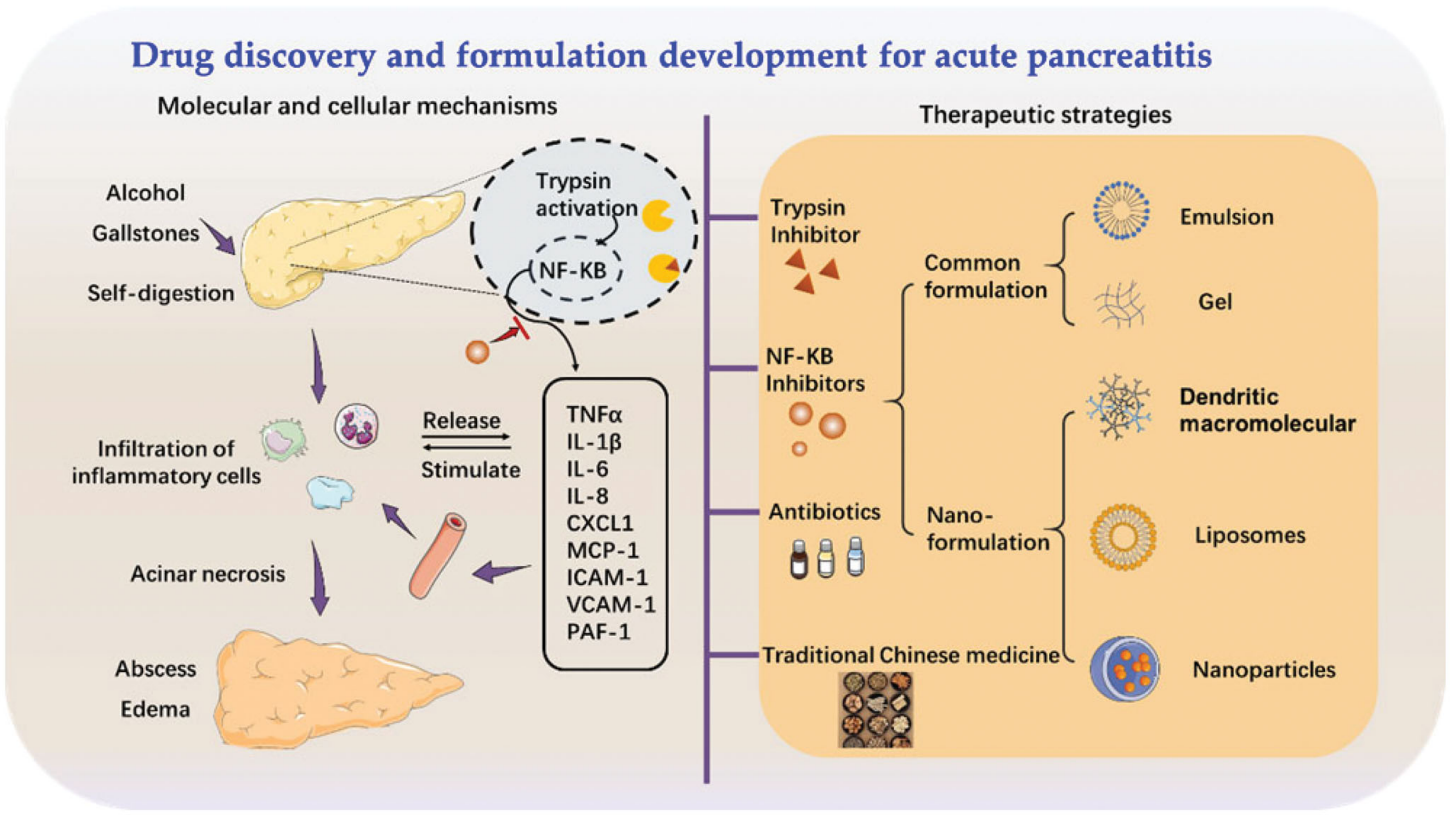
The cellular events in acute pancreatitis and therapy strategies.

Nanoparticulate systems have become prevalent drug delivery systems over the past few decades. Novel drug delivery systems can be engineered to improve bioavailability, control the drug release rate, increase the accumulated concentration at the desired site, and reduce the systemic toxicity of the drugs. Recently, nanoparticulate strategies that enclose therapeutics drugs in nano-sized preparations revolutionized the treatment of acute pancreatitis. With the find of new target drugs, nanoparticulate could provide a platform to precisely deliver into the inflammatory lesions and might extend its application in pancreatitis treatment. Additionally, some nanoparticles with pharmacological activities have reported attenuating the pancreatic inflammation. Herein, in this review, we will briefly introduce the involved pathogenesis and underlying mechanisms of acute pancreatitis, as well as the traditional Chinese medicine as the new drug option (Figure 1). Most of all, we will summarize the use of nanoparticulate strategies to reduce inflammation and potentially prevent the further development of acute pancreatitis, with an emphasis on the bifunctional nanoparticles that act as both drug delivery carriers and therapeutics.

## 2. Molecular and cellular mechanisms of acute pancreatitis

**Figure 2.**
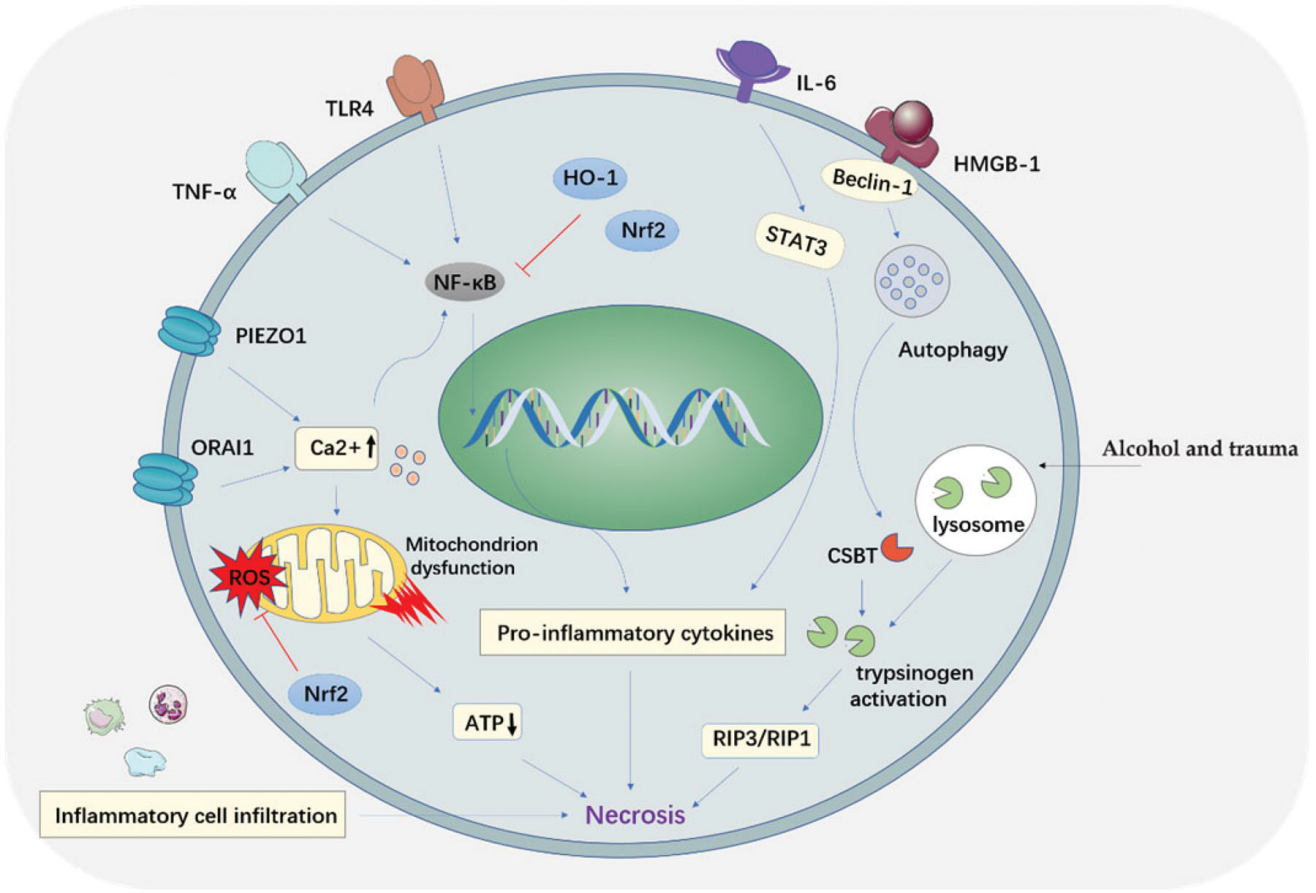
Cellular events and potential therapeutic targets for acute pancreatitis.

The etiology and pathogenesis of acute pancreatitis have been widely studied. Complex cellular events, e.g. premature trypsinogen activation, pathological calcium overload, pancreatic microcirculation disorders, activation of NF-κB, infiltration of leucocyte cells, and impaired autophagy, have been reported to participate in the pathogenesis of acute pancreatitis. Etiologies, including bulbosity and bile duct damage, played a critical role in triggering cell events in acute pancreatitis. Understanding these molecular and cellular mechanisms in acute pancreatitis will help us to identify potential drugs those may benefit acute pancreatitis treatment in the future (Figure 2).

### 2.1. Premature trypsinogen activation

Premature trypsinogen activation is an important pathological cellular event that can lead to acinar cell necrosis. Trypsin exists as an inactive enzyme granule in the normal physiological state. In acute pancreatitis, the release of zymogen granule is checked and activated by the cellular lysosome, thus leading to the intracellular activation of digestive enzymes, e.g. trypsin. Additionally, the elevated tissue edema, serum amylase level, lipase level, inflammatory cell infiltration, and acinar cell death were closely related to the increased trypsin activity (Zhan et al., 2019).

Various stimuli, e.g. alcohol, pancreatic ductal obstruction, and trauma, could trigger the fusion between zymogen granules and lysosome, a process called colocalization of lysosomes with pancreatic digestive enzymes. This process is involved with lysosomal cathepsin B (CTSB), a critical lysosomal enzyme. The over released CTSB can cause trypsinogen activation and acinar cell necroptosis. The protective effect of inhibiting trypsinogen activation in acute pancreatitis development was demonstrated in CTSB gene knockout mice (Halangk et al., 2000). Additionally, the activated trypsinogen could attack the neighboring acinar cells and cause necroptosis, which mediated by the receptor-interacting protein kinase (RIP) (Figure 2) (He et al., 2016). Therefore, RIP3/RIP1 could also act as a therapeutic target to protect acinar cells from the enzyme attack in the therapy of acute pancreatitis.

### 2.2. Calcium overloading and mitochondrial dysfunction

Calcium is involved in the activation of trypsinogen, mitochondrial polarization and acinar cell apoptosis. The overloaded calcium ion could induce the injury to pancreatic acinar cells and necrosis by mediating mitochondrial dysfunction (Figure 2). Calcium channel protein1 (ORAI1) was the crucial way for calcium transport. ORAI1 inhibitors were confirmed effective in the therapy of acute pancreatitis (Wen et al., 2015). Latest research further confirmed that TRPV4 channel opening was responsible for sustaining calcium concentration elevation in acute pancreatitis after calcium-permeable ion channel (Piezo1) stimulation (Swain et al., 2020).

Additionally, studies have demonstrated that calcium ion concentration can activate nuclear factor (NF)-κB signaling pathway, secondary triggering a cascade of inflammatory responses (Steinle et al., 1999; Tando et al., 1999). Latest research pointed out that Na^+^-Ca^2+^ exchanger 1 (NCX1) ion promoter was bonded with NF-κB to govern calcium overloading in acute pancreatitis development. Thus, calcium overloading and NF-κB activation might be a mutually interacted pattern.

Mitochondrial dysfunction was the key organelle disorder in acute pancreatitis development. Acute pancreatitis caused a nonselective opening of channel in the mitochondrial membrane and consequent ATP consumption (Forsmark et al., 2018). Mitochondrial permeability transition pore inhibition provides a promising approach to suppress mitochondrial dysfunction, which was beneficial for acute pancreatitis treatment. Mitochondrial transition pore inhibitor, 3,5-seco-4-nor-cholestan-5-one oxime-3-ol (TRO40303) and N-methyl-4-isoleucine cyclosporin (NIM811) were proved to be beneficial to overcoming acute pancreatitis by inhibiting mitochondrial dysfunction and reducing cell necrosis (Javed et al., 2018; Tóth et al., 2019). Thus, inhibiting mitochondrial transition pore might provide new sight for acute pancreatitis treatment.

### 2.3. Microcirculation disturbance

Microcirculation disturbance is another pathophysiologic mechanism contributing to the progression of acute pancreatitis. Acute pancreatitis occurs often accompanied by multiplying secretion of inflammatory cytokines, resulting in the pancreas microcirculation disorder. Considerable cell damage occurred in the vascular endothelial cells while exposed to NO, slow excitation peptide (BK), platelet-activating factor (PAF), etc. Abnormal hemorheology also promoted the development of acute pancreatitis from edema to necrotizing. Platelet-activating factor receptor antagonists (PAF-RAs) was found to significantly reduce local and systemic pathogenesis in acute pancreatitis (Chen et al., 2008). The early microcirculation disorder of the pancreas can directly or indirectly damage the pancreas, resulting in ischemia injury as well as inflammatory reactions. Correcting or modulating the microcirculation disorder is beneficial for the invert of acute pancreatitis in clinic.

### 2.4. Inflammation in acute pancreatitis

#### 2.4.1. Activation of NF-κB

**Figure 3.**
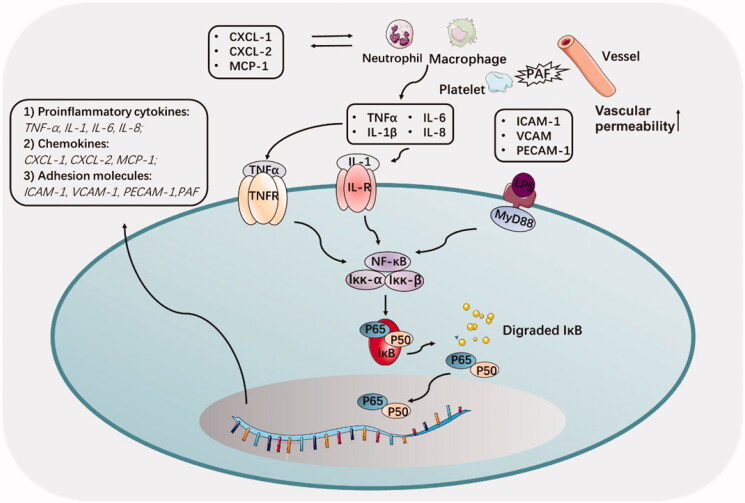
The regulation of NF-κB signaling pathway and involved cytokine and adhesion molecules in acute pancreatitis. Inhibition of NF-κB reduces the induction of pro-inflammatory cytokines, chemokines, and adhesion molecules.

NF-κB remains dormant in the cytoplasm through interaction with the IkB family (inhibitory proteins), which limits the translocation of NF-κB into the nucleus and its binding to DNA. In acute pancreatitis, inflammatory cytokines, e.g. tumor necrosis factor (TNF)-α and interleukin 1 (IL1), could combine with receptors and activate the IkBa kinase, which released the NF-κB from the cytoplasm NF-κB/IkB complex and forming a p50/RelA dimers (Jakkampudi et al., 2016) (Figure 3). Then the p50/RelA dimers could transfer into the nucleus, and reacted with target gene elements by p50 subunits to launch a target gene expression, including promoting apoptosis factor Bax, and TNF-α (Jakkampudi et al., 2016).

Many studies have shown that activation of NF-κB is an early and central event in the progression of inflammation of acute pancreatitis. Duan et al. demonstrated that the receptor-interacting protein kinase 1(RIPK1)/NF-κB/aquaporin 8 (AQP8) axis might serve as a potential regulatory pathway to inhibit acinar cell necrosis in early acute pancreatitis (Duan et al., 2019). The latest study proved that C1q/tumor necrosis factor-related protein 3 (CTRP3) exerted protective effects in acute pancreatitis via suppressing silent information regulator 1 (SIRT1)/NF-κB/p53 axis (Lv et al., 2020).

Nucleotide-binding oligomerization domain (NOD) receptor and Toll‐like receptor 9 (TLR9) may also involve in the regulation of NF-κB expression and the oxidation/antioxidation balance, which can be a useful therapeutic target for SAP (Yan et al., 2017). It has been demonstrated that nearly all pathways leading to NF-κB activation could be blocked by treatment with a variety of antioxidants, including N-acetyl-L-cysteine (NAC), glutathione (GSH), and thioredoxin (Mercurio & Manning, 1999). In recent years, a large number of active drugs have emerged to improve therapy for acute pancreatitis by inhibiting the NF-κB signaling pathway in experiments (Cen et al., 2016; Tao et al., 2019). Bilirubin is a bioproduct, and possessed potent antioxidant and anti-inflammatory properties (Yao, Jiang, et al., 2019; Yao, Jiang, 2019; Yao, Huang, 2020; Yao, Sun, 2020). Our previous study also indicated that bilirubin could inhibit the NF-κB mediated pro-inflammatory signaling pathway, and significant reduced the progression of acute pancreatitis in a rat model (Wang et al., 2016).

#### 2.4.2. Role of pro-inflammatory cytokines

Pro-inflammatory cytokines, such as TNF-a, IL-1β, IL-6 and IL-8, play a wicked role in the inflammatory pancreas, not only trigger other signaling pathways but also directly accelerate the course of the inflammation (Duan et al., 2019). A study showed that these pro-inflammatory cytokines were released and then interacted with peripheral blood mononuclear cells (PBMC), leading to acinar cell apoptosis (Jakkampudi et al., 2017). Among these pro-inflammatory cytokines, TNF-α needed to be emphasized by its evocable large recruitment of macrophages to inflamed sites in AP. In addition, TNF-α can activate damaged endothelial cells to secrete vascular adhesion factor (PECAM-1), intercellular adhesion factor (ICAM-1), and selectin (Malleo et al., 2007). Besides, IL-6 was always considered to participate in acute pancreatitis by facilitating neutrophils infiltration. Latest research provided novel cellular signal pathway that IL-6 promoted TMEM16A expression via activating IL-6R/STAT3 signal to facilitate pathogenesis of acute pancreatitis (Wang et al., 2020).

Suppressing these pro-inflammatory cytokines secretion has been recognized as a feasible strategy for pancreatitis treatment. HO-1 is the cytoprotective enzyme that controls inflammatory responses and redox homeostasis (Li et al., 2018). Induction of HO-1 pathway in early SAP could modulate the systemic inflammatory response and prevent liver injury via augmentation of IL-10 and inhibition of TNF-α (Zhang et al., 2017). Additionally, TNF-α antibodies and receptor blockers have also shown local protective effects in animal studies of acute pancreatitis, but no clinical trials have been conducted so far (Malleo et al., 2007). Tocilizumab, the IL-6 receptor antibody has been proven effective in acute pancreatitis (Chen et al., 2016), and clinically used in patients with active rheumatoid arthritis (Bae & Lee, 2018).

Besides, IL-15 is a cytokine that usually released by epithelial cells and holds the immunomodulation properties by stimulating the production of T cells and natural killer cells. However, a recent study demonstrated that IL-15 treatment protected mice from the cerulein-induced chronic pancreatitis pathogenesis by regulating fibrosis and inflammation (Manohar et al., 2018). Although with no direct experimental evidence, current data indicated that IL-15 immunotherapy might be a possible and potential strategy for restricting the progression of fibrosis in pancreatitis.

#### 2.4.3. Infiltration of inflammatory cells

Acute pancreatitis was commonly characterized by high inflammatory cell infiltration in the pancreas (Werner et al., 1998). Inflammatory cell infiltration is an early and critical event in the initial development of AP, the underlying mechanism of which was always interpreted as an inciting action by chemokines secreted by the acinar cell, parenchymal cell, and mesenchymal cell (Vonlaufen et al., 2007). A 50% increase in membrane potential was observed in CXCL10-treated acinar cells, as well as increased expression of cytochrome C, caspase 9/3 activation, and ATP consumption (Singh et al., 2010). CXCL16 contributed to the development of acinar cell necrosis through the induction of CCL9 and subsequent neutrophil infiltration (Sakuma et al., 2018). The recent study verified that HO-1 could suppress neutrophil infiltration in inflamed pancreas via CXCL2 inhibition (Bae et al., 2019). This evidence represented the crucial role of chemokines in the inflammatory cells trafficking.

Neutrophils, the key participants in the AP, has to be addressed here, which could release myeloperoxidase (MPO) and ROS into the interstitium to exert toxicity (Yang et al., 2015). The activated neutrophils also contributed to the neutrophil extracellular traps (NETS) generation, involved in the acute pancreatitis progression by worsening inflammation and promoting pancreatic duct obstruction (Murthy et al., 2019). C-Abelson (c-Abl), the crucial kinase in the NETS formation, might serve as a new target in the acute pancreatitis treatment, as evidenced by the beneficial effect of c-Abl kinase inhibitor (GZD824) in L-arginine-induced pancreatitis by decreasing amylase level, pro-inflammatory cytokines level, neutrophil infiltration as well as acinar cell necrosis (Madhi et al., 2019).

### 2.5. Oxidative stress

Studies have highlighted the role of oxidative stress in the acute inflammatory response, particularly in the pancreatic injury associated with acute pancreatitis (Tsai et al., 1998; Bopanna et al., 2017). Oxidative stress presents as the unbalance of free radicals and antioxidants, might serve as a malicious propeller in the development of acute pancreatitis. On the one hand, ROS can induce migration of leukocytes and a variety of inflammatory cytokines, chemokine secretion. On the other hand, oxidative species such as H_2_O_2_ and α, β-unsaturated aldehydes directly served as second messengers in the inflammation (Que et al., 2010). Indeed, the ROS induced DNA injury could be reflected by the increased levels of lipid peroxidation, myeloperoxidase activity, and protein carbonyls in patients with SAP (Kiselyov & Muallem, 2016). Additionally, nuclear factor erythroid 2-related factor 2 (Nrf2), a master regulator of the adaptive cellular response to oxidative stress, has been proved to mitigate acute alcohol-induced hepatic and pancreatic injury in mice by regulating antioxidant response. Our recent study also indicated that up-regulation of Nrf2/HO-1 pathway could help to reduce the severity of acute pancreatitis (Wang et al., 2016).

One well-known mechanism of oxidative stress-induced injury in acute pancreatitis is the activation of the NF-κB signaling pathway. Recent attention has been put on the relationship between ROS and calcium signaling since Ca^2+^ homeostasis is sensitive to cellular redox status (Brookes et al., 2004). It is worth mention that many vital proteins, such as orai1 (Malleo et al., 2007) and plasma membrane Ca^2+^-ATPase pump (Bruce & Elliott, 2007), might have participated in the redox regulation and Ca^2+^ level.

### 2.6. Autophagy

Autophagy, the lysosome-mediated degradation pathway, participated in the secretion and degradation of protrypsin granules in pancreatic acinar cells. In vitro and in vivo data indicated that normal autophagy function in acute pancreatitis was disrupted, leading to abnormal activation of trypsinogen (Malla et al., 2019), endoplasmic reticulum stress, and mitochondrial dysfunction, leading to the necrosis of acinar cells (Biczo et al., 2018). A recent study demonstrated that autophagy inhibitor chloroquine could inhibit the NET formation and decrease the severity of acute pancreatitis (Murthy et al., 2019). Therefore, therapeutic approaches that target restoration of autophagy can serve as a promising option for acute pancreatitis therapy.

### 2.7. High-mobility group box-1(HMGB-1)

High-motility group box protein 1 (HMGB1) is recognized as an important predictor for the persistence and further development of pancreatitis (Arriaga-Pizano et al., 2018). HMGB-1 is an advanced inflammatory factor that is usually produced in the late stage of inflammation and keep at a high level for a while, which is different from TNF-a, IL-6, and other inflammatory factors. Research showed that HMGB1 level in pancreatic tissues is abnormally increased in experimental SAP model (Li et al., 2016). Additionally, HMGB-1 participated in the intestinal mucosal damage and dysbacteriosis, which often occurs in the SAP induced multiple organ inflammation (Chen et al., 2017). Inhibition of HMGB-1 played a protective role in intestinal mucosal barrier dysfunction by significantly reducing the serum levels of many pro-inflammatory cytokines, e.g. IL-1β, IL-6, and TNF-α. HMGB-1 inhibition attenuated bacterial translocation and decreased mortality in murine acute pancreatitis model (Huang et al., 2019).

Furthermore, HMGB-1 plays an important role in autophagy. MiR-141, one type of non-coding RNAs, was confirmed to block the autophagosome formation via the HMGB1/Beclin-1 pathway, resulting in the inflammatory pancreas (Zhu et al., 2016). Therefore, the intervention of HMGB-1 and autophagy appears to be a promising alternative option for the gene therapy of pancreatitis.

## 3. Therapeutic drugs for acute pancreatitis

Acute pancreatitis is not a simple inflammatory reaction, but rather more complicated, which is characteristic of early pancreatic enzymes activation in the pancreas, causing digestion, edema, bleeding, and even necrosis of pancreatic tissues. The clinical manifestations of acute pancreatitis, especially SAP, are dangerous and life-threatening while the prognosis is poor. The key to acute pancreatitis treatment remains the intervention of disease development.

### 3.1. Commonly used drugs in clinic

In clinic, acute pancreatitis intervention within the first 72 h is crucial. The approaches vary, includes fluid resuscitation, analgesia, and early enteral feeding, focusing on the improvement of intestinal mucosal barrier function and reducing complications. However, no medications are used to treat acute pancreatitis specifically, and clinical treatment outcomes are far from satisfactory (Andersen et al., 2018). The existing clinical drug treatment plan is mainly aimed at reducing pancreatic secretion, relieving spasm and pain, and anti-infectious treatment (Table 1). However, the discrepancy about the beneficial effect of these medications on pancreatic injury still exists. Many of these well-reported medications showed heterogeneous results in the clinic, which raised many concerns (Lyu et al., 2019). Glucocorticoids were also found to show efficacy with ascitic fluid and pancreas histopathology (Yu et al., 2014). To date, the use of antibiotics is generally recommended when the pancreatic necrotic infections occur, otherwise it remains strictly restricted. The lipophilicity of antibiotics that also limited the blood pancreas barrier (BPB) transport efficiency and compromised the pancreatitis treatment.

**Table 1.** List of common drugs in the acute pancreatitis treatment.

Types	Drugs	Pharmacological activities against pancreatitis	Applications
Analgesics	Atropine	Relieve spasm and reduce the pancreatic secretion by blocking the extrusion of zymogen granules.	MAP to SAP
	Meperidine	As synthetic opioid narcotic analgesic for the relief of severe pain.	MAP to SAP
Pancreatic secretory trypsin inhibitor	Pantoprazole	H-K-ATPase inhibitor and suppress gastric acid secretion	MAP to SAP
	Ulinastatin	Suppress many serine proteases, e.g. trypsin, chymotrypsin, kallikrein, hyaluronidase and granulocyte elastase; Stabilize lysosome membrane and inhibit lysosome function; Inhibit calcium influx of the cell transport system; Reduce the production of many pro-inflammatory cytokines, e.g. TNF-α and IL-1β.	AP (including traumatic, postoperative and endoscopic retrograde pancreatitis) MODs caused by AP
	Gabexate	Acting as a synthetic low molecular weight protease inhibitor.	MAP
	Somatostatin	Inhibit the secretion of the pancreas; Protect the intestinal barrier; Reduce intraperitoneal infections; Relaxes the Oddi sphincter and promote bile and pancreatic drainage.	AP (including traumatic, postoperative and endoscopic retrograde pancreatitis)
Gram-negative bacteria antibiotics	Ciprofloxacin	It has a wide antibacterial spectrum and strong antibacterial effect.	Endogenous infection and secondary infections in biliary AP
	Cefoperazone	It has a wide antibacterial spectrum and strong antibacterial effect.	
	Aztreonam	It has a high antibacterial activity against most aerobic Gram-negative bacteria.	
Anaerobes antibiotic	metronidazole	It possesses significant antimicrobial activity against several obligate anaerobes.	To prevent infectious complications in pancreatitis.

### 3.2. Potential traditional Chinese medicine

Traditional Chinese medicine has been used in the clinical intervention of acute pancreatitis in Asia. While most modern medicines only provide endeictic relief in pancreatitis, traditional Chinese medicine has been proven to be more effective in adjusting the body homeostasis and alleviating the clinical symptom. For example, Chaiqin Chengdi Decoction has been reported to significantly improve the intestinal paralysis in clinic, which could effectively promote the gastrointestinal motility and benefit the recovery of gastrointestinal function in patients with acute pancreatitis (Pan et al., 2017). Another study demonstrated that Chaiqin Chendi Decoction could upregulate the expression of endoplasmic reticulum Ca^2+^-ATPase in pancreatic tissues, reduce intracellular calcium overload and relieve pancreatic tissue lesions (Bae et al., 2019). Clinical study also indicated that Chaiqin Chendi Decoction serum notably decreased matrix metalloproteinase nine (MMP 9) level in patients with SAP (Guo et al., 2013), while MMP 9 was positively correlated with the pathological score.

Many traditional Chinese medicines have been investigated in the animal model and achieved encouraging results. Danshen injection exerted protective effects on the intestinal mucosa of SAP by inhibiting apoptosis and downregulating NF-κB signaling pathway (Zhang et al., 2010). Qingyi Decoction ameliorated acute biliary pancreatitis by inhibiting Gpbar1/NF-κB/p-RIP (Zhang, 2019), which improved intestinal myoelectrical activity and intestinal transit in odium deoxycholate (SDOC) induced acute pancreatitis rats model. Sheng-Jiang powder ameliorated obesity-induced pancreatic inflammatory injury in rats (Miao et al., 2018). Dachaihu Decoration could ameliorated the L-arginine-induced pancreatitis via inhibiting the infiltration of macrophages and decreasing the gene expression of macrophage inflammatory protein 1α (MIP-1α), IL-6 and monocyte chemoattractant protein-1 (MCP-1) (Duan et al., 2017).

Innovative administration has made Chinese herbs easier for clinical application. Researchers have improved the clinical effect and shortened the course of treatment of MAP by feeding compounds from Chinese medicine concentrated decoction (Joubert et al., 2008). In addition, Rhubarb and Peony Decoction combined with nursing intervention for patients with acute pancreatitis could effectively relieve abdominal pain, abdominal distension, nausea, vomiting and other clinical symptoms in a randomized controlled trial (RCT) (Zhou et al., 2016).

The extraction and refinement of Chinese medicine also have good therapeutic outcomes in acute pancreatitis. Phenolics compounds such as curcumin (Yu et al., 2018; Siriviriyakul et al., 2019), resveratrol (Wang et al., 2017), honokiol (Weng et al., 2012), caffeic acid phenyl ester (Buyukberber et al., 2009), ellagic acid (Yılmaz et al., 2016) and sesamol (Chu et al., 2012) have been reported to exert protective effect in acute pancreatitis. Many quinones ingredients from the DaChengQi Decoction, e.g. emodin, rhubarb and shikonin, also have been reported to be effective against pancreatitis (Sun et al., 2020). As shown in Table 2, we have summarized the natural derived bioactive compounds that have been demonstrated to be effective against acute pancreatitis in the experimental animal models in the recent five years. Besides, some bioactive compounds that extracted from the traditional Chinese medicine have been formulated and applied to treat acute pancreatitis in China. For example, danhong injection can significantly improve blood rheology, increase pancreatic blood flow, and improve pancreatic microcirculation (Liu et al., 2017). Xuebijing injection can effectively relieve the clinical symptoms of acute pancreatitis and shorten the length of hospital stay under conventional treatment (Zheng et al., 2015). Shenfu injection has shown a great protective effect in acute pancreatitis but still needed to be verified in clinic (Yao et al., 2014). Multi-center and high-quality RCTs with large sample sizes are still needed to provide evidence for the therapeutic efficacy and safety of these herbal bioactive compounds for clinical guidelines.

**Table 2.** Effective naturally derived drugs in experimental treatment of pancreatitis in the last 5 years.

Drugs	Characteristics	Animal model	Indications	Cellular mechanism	Ref
Isoliquiritigenin	Flavonoid monomer	Caerulein, mice	MAP	Modulate the Nrf2/HO-1 pathway.	
Emodin	Active ingredients of Chinese medicine	Sodium taurocholate, rats	SAP	Inhibit the P2X7/NLRP3 pathway.	(Zhang et al., 2019)
SRT1720	SIRT1 activator	Sodium taurocholate, rats	SAP	Inhibit the NF-κB pathway.	(Shi et al., 2018)
Calycosin	Isoflavone isolated from Radix astragali	Caerulein, mice	SAP	Inhibit the NF-κB and p38-MAPK pathway.	(Miraghazadeh & Cook, 2018)
Curcumin	Thiazolidinediones	Sodium taurocholate, rats	SAP	Suppress TRAF1/ASK1/JNK/NF-κB pathway.	(Yu et al., 2018)
Ligustrazine	Active ingredient of ligusticum chuanxiong	Caerulein, rats	SAP	Suppress p38 and Erk pathway.	(Chen et al., 2016)
Chemerin	Adipokine, chemoattractant for the immune cells	Caerulein, rats	MAP	Inhibit the NF-ΚB pathway.	(Jaworek et al., 2019)
Visnagin	A phytochemical isolated from Ammi visnaga	Caerulein, mice	MAP	Activate the Nrf2/ARE pathway and inhibit the NF-кB pathway.	(Pasari et al., 2019)
Menadione	Vitamin K3	Caerulein, mice	MAP	Inhibit hydrogen sulfide and substance P via the NF-кB pathway.	(Amiti et al., 2019)
Dexamethasone	Glucocorticoid	Caerulein, rats	SAP	Suppress the expression of NF‐κB/p65 and HMGB1.	(Xu et al., 2018)
Adiponectin	Active peptide from adipose	Cerulean, rats	MAP	Reduce the activity of the NF-κB pathway.	
β-Arr1	Mediators of G protein-coupled receptor	Caerulein, mice	MAP	Suppress the activation of NF-κB/p65 pathway.	
Baicalin	Flavonoid	Sodium taurocholate, rats	SAP	Down-regulate protein kinase D1 and NF-кB protein expressions.	(Qian et al., 2018)
Melatonin	Amine hormone	Taurocholate, rats	SAP	Inhibit the activation of p38 MAPK and NF‐κB pathway.	(Chen et al., 2018)
Pioglitazone	Thiazolidinediones	Taurocholate, rats	SAP	Inhibit the activation of p38 MAPK and NF‐κB pathway.	(Hai et al., 2018)
Docosahexaenoic Acid	An ω-3 fatty acid	Cerulein, rats	MAP	Suppress the activation of NF-κB and PKCδ pathway.	(Jeong et al., 2017)
Luteolin	Bioactive component of Reseda odorata	Cerulein and lipopolysaccharide, rats	SAP	Exert HO-1-mediated anti-inflammatory and antioxidant effects.	(Xiong et al., 2017)
Sulforaphane	A natural organosulfur antioxidant	Cerulean, mice	MAP	Modulate Nrf2-mediated oxidative pathway and NLRP3/NF-κB inflammatory pathways	
Artesunate	Artemisinins	Sodium taurocholate, rats	SAP	Down regulate the TLR4/NF-κB pathway	(Cen et al., 2016)
Withaferin A	Ashwagandha Extract	Cerulein,	MAP	Relieve ER stress and the NLRP3 inflammasome via NF-κB pathway	
Flavonoid C1	Flavonoid from Coreopsis tinctoria	Taurocholate, rats	SAP	Regulate Nrf-2/ARE-mediated antioxidant pathway	(Du et al., 2018)
Naringenin	Flavonoid	Caerulein and L-arginine, mice	MAP&SAP	Regulate NLRP3 and Nrf2/HO-1 pathway	
dh404	Synthetic Triterpenoid	Caerulein and L-arginine, mice	MAP&SAP	Activate Nrf2 pathway	(Robles et al., 2016)

## 4. Formulation approaches for acute pancreatitis

With the depth understanding of pathogenic mechanisms of pancreatitis, it is pivotal to inhibit the secretion of pancreatic enzymes and mitigate the wicked inflammatory microenvironment. But it is still challenging to deliver the therapeutics to the pancreas, which was characterized by lower pH, overexpressed pancreatic enzymes, impaired blood vessels, and elevated oxidative stress. Several issues have to be addressed to improve delivery efficiency. First, how to ensure the therapeutics could selectively target the pancreas after administration. Secondly, how to cross the BPB, which provided a difficult barrier for most anti-inflammatory drugs and resulted in compromised drug concentration in the pancreas (Choi et al., 2018). Moreover, most trypsin inhibitors for pancreatitis treatment are peptides, which often undergo a short half-life during the circulation in clinic. In the end, the harsh microenvironment of the pancreas in patients with acute pancreatitis also influenced the drug release and pharmacological activities.

In this section, we introduced recent progress of formulation approaches for acute pancreatitis. Formulation strategies were applied to enable the delivery of a therapeutic drug in the body and improve its efficacy and safety by optimizing the pharmacokinetics and targetability to alleviate the pancreatitis development. Here, we emphasized the nanotechnology and biomaterials-based formulations. Nanoparticles could prolong the circulation time and selectively accumulated at the inflamed tissue. Also, these newly developed nanoparticulate formulations could be designed to target the physical characteristics of inflamed pancreas, e.g. weakly acidity and abundant digestive enzymes. Some recent studies also reported neutrophil cell derived membrane camouflaged nanoparticles could disguise as natural neutrophil and actively recruited into the inflamed pancreas after intravenous injection. Other than utilizing the cellular mechanisms of acute pancreatitis for improved delivery efficiency, some nanoparticles could exert drug actions by directly inhibiting the cellular events that contributed to the acute pancreatitis development. For example, polyamidoamine (PAMAM) dendrimers could contributed to the inhibition of NF-κB nuclear translocation in macrophages and reduction in inflammatory cells. Some metal nanomaterial, cerium oxide nanoparticles (Ce_2_O_3_ NPs) and yttrium oxide nanoparticles (Y_2_O_3_ NPs), could attenuate the oxidative stress by inhibiting the Nrf2/NF-κB pathway. Besides, biomimetic carbon monoxide delivery system can suppress systemic inflammation through inhibiting neutrophil infiltration and HMGB-1 production.

Hence, in this part, we will not only summarize the common formulation methods to deliver therapeutics for acute pancreatitis such as chemical modification, gel and emulsions, but also focus on the recent nanoparticulate approaches that potentially change the therapeutic outcomes and their involved mechanism in the acute pancreatitis management.

### 4.1. Nanoparticulate formulation strategies for acute pancreatitis

Nanotechnology has gained considerable attention as a drug delivery carrier (Kou et al., 2013, 2017a, 2017b, 2018a, 2018b, 2018c). One of the most well-known benefits of nanomedicine is their selective accumulation by the enhanced permeability and retention (EPR) effect at tumor sites (Yao et al., 2018; Kou et al., 2019; Yao et al., 2019, 2020; Zhao et al., 2020). Recent studies have indicated that similar EPR effect also occurred in the inflammatory lesion, that analogous nanoparticles within certain size range could accumulate in the inflamed site through the effect of extravasation across leaky vasculature and subsequent inflammatory cell-mediated sequestration, so-called ELVIS effect (Gong et al., 2019). In the last decades, the ELVIS effects have been exploited for passive targeting of nanoparticles into the inflamed site with increased delivery efficiency and reduced systemic toxicity. As a delivery carrier for acute pancreatitis therapy, nanoparticles have multiple advantages over traditionally formulation methods, including (1) increasing the drug stability in the circulation; (2)protecting the drug from biological clearance and prolonging the half-life; 3) selectively accumulating at the inflammatory site via ELVIS effect; (4) easily to modify with functional motifs to achieve controlled release in the inflammatory site by taking advantages of the pathological characteristics, e.g. pH, ROS, and trypsin. Additionally, there have emerged various potential nanoparticles with intrinsic therapeutic characteristics for the pancreatitis treatment, such as PAMAM dendrimers and metal nanomaterials. In this section, we will introduce the nanoparticles to either delivery the therapeutics or exert pharmacological activities by itself in the treatment of acute pancreatitis.

#### 4.1.1. Anti-inflammatory PAMAM dendrimers

**Figure 4.**
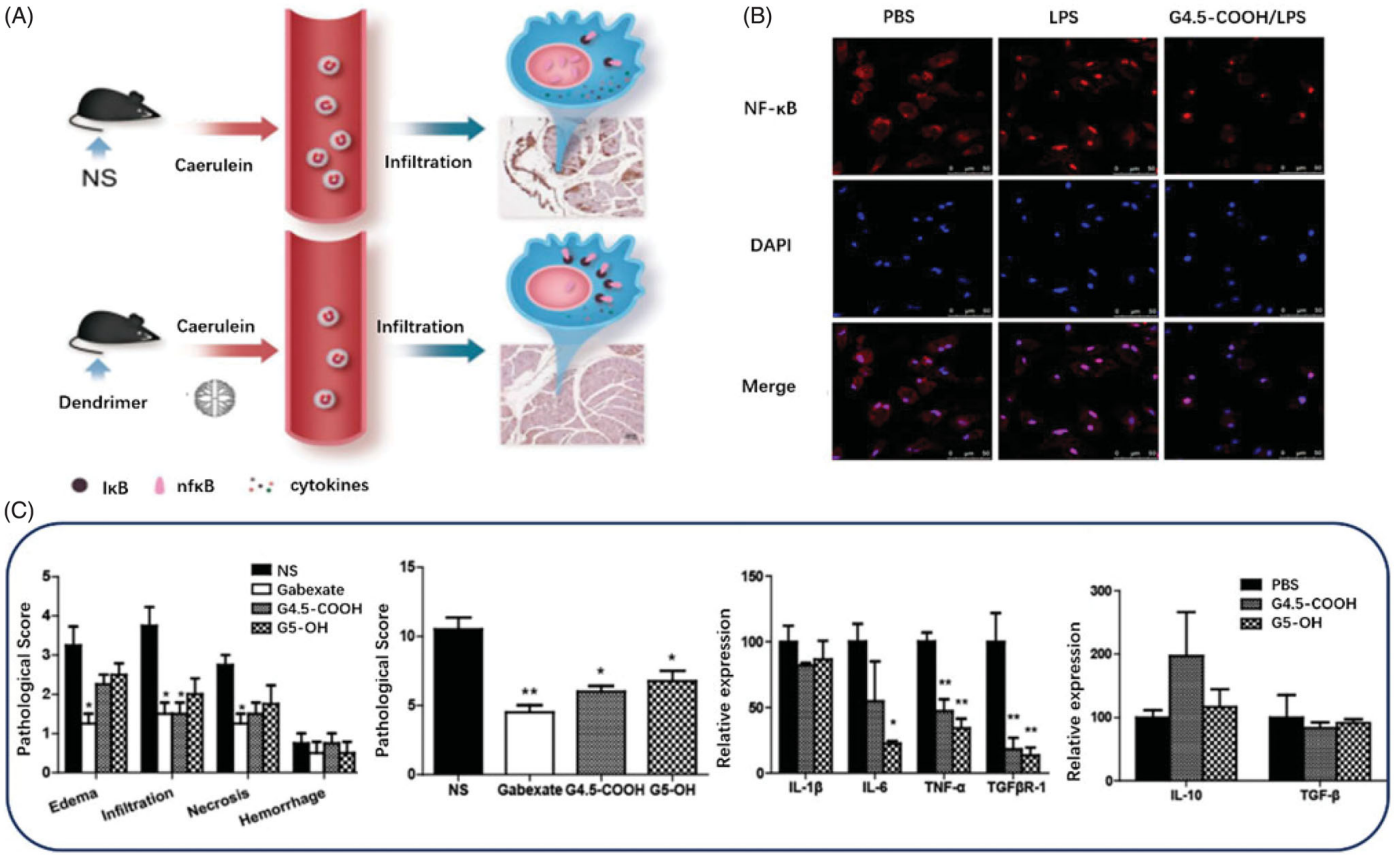
(A) Schematic diagram of treatment of PAMAM Dendrimer in acute pancreatitis. (B) NF-κB nuclear translocation in macrophages investigated by confocal microscopy. Alexa Fluor labeled NF-κB protein is shown in red, and DAPI-labeled cell nuclei are shown in blue. (C) The pathological score, pro-inflammatory cytokines level and anti-inflammatory cytokines level in G4.5-COOH and G5-OH PAMAM treated rats (Tang et al., 2015).

PAMAM dendrimers that composed of diamine (usual ethylenediamine) nuclear and branched surface groups have been widely used as suitable drug delivery carriers for genes and poorly water-soluble molecules in a variety of disease models linked with oxidative stress (Lai et al., 2019; Mahmoudi et al., 2019). PAMAM dendritic macromolecule is one of the most promising nanocarriers with a spherical and flexible structure, fine and uniform size, the sufficient number of surface groups for functional modification. The previous research has used PAMAM dendrimers as intrinsic therapeutics for multiple inflammatory diseases, like ischemia/reperfusion injury. Matsuura et al. modified PAMAM dendrimers with l-cysteine and l-serine and achieved dominant renal distribution in a renal ischemia/reperfusion injury animal model (Matsuura et al., 2018a, 2018b). Their studies first confirmed that PAMAM dendrimers efficiently scavenge ROS and prevent renal ischemia/reperfusion injury. Inspired with the nanoparticulate structure and intrinsic anti-inflammatory properties, the scientist also studied the potentials of PAMAM dendrimers to treat acute pancreatitis as presented in Figure 4 (Tang et al., 2015). In this study, they investigated the protective effect of G4.5-COOH and G5-OH PAMAM dendrimers against pancreatic impairment in caerulein-induced acute pancreatitis model (Tang et al., 2015). This very initial study confirmed that compared to G5-OH, G4.5-COOH exerted a more substantial protective effect against pancreatitis. The therapeutic mechanism of PAMAM dendrimers mainly involved the inhibition of NF-κB nuclear translocation in macrophages and reduction in inflammatory cells in the pancreas and peripheral circulation.

While they could exert anti-inflammatory properties itself, PAMAM dendrimers also have been considered as a versatile anti-oxidant delivery system to achieve synergistic effects. For example, glutathione is a potent endogenous antioxidant and capable of preventing damage to important cellular components caused by various types of ROS, e.g. free radicals, peroxides, and lipid peroxides. PAMAM-GSH conjugate has been synthesized to delivery GSH using the PAMAM nanoplatform, and demonstrated to effectively reduce the intracellular levels of ROS in PC12 cells (Sun et al., 2016). Therefore, it is reasonable to assume that PAMAM-GSH conjugates might serve as a useful alternative for acute pancreatitis in the future.

While various antioxidant enzymes depletion, including superoxide dismutase (SOD), catalase, and GSH, often occurred in pancreatitis, it is acknowledged that the replenishment of antioxidant agents, e.g. NAC, have been recognized as a feasible idea for acute pancreatitis therapy (Atayoglu, 2016). However, a recent double-blind placebo (PL)-controlled randomized pilot study indicated the simple supplementation with antioxidant to patients obtained no additional therapeutic benefits on endocrine and exocrine function, fibrosis progression, and the severity of oxidative stress and inflammation (Singh et al., 2019). This result was consistent with the clinic treatment of acute pancreatitis that NAC was more often used combined with anti-inflammatory agents rather than alone. For example, a new study suggests that hybrid nonsteroidal anti-inflammatory drugs and N-acetylcysteine therapy are effective for prevention of post-retrograde cholangio pancreatography (ERCP) pancreatitis (Ma et al., 2019; Pavel et al., 2019). Therefore, it is of clinic importance to use combination of antioxidants and anti-inflammatory agents to achieve better therapeutic outcomes. For PAMAM dendrimer system which holds the intrinsic anti-inflammatory properties, PAMAM could either encapsulate or conjugated to antioxidants and act as nanotherapeutics with double-duty.

Recent research also reported a ROS responsive PAMAM derivatives, poly(amidoamine)-N-(4-boronobenzyl)-N, N-diethyl-2-(propionyloxy)ethan-1-aminium (PAMAM-(B-DEAEP)), as a gene vector (Li et al., 2019). The surface charge of PAMAM-(B-DEAEP) could vary from negative to positive under the elevated ROS levels, including cancerous tissues and inflammatory tissues. Given the important role of ROS in the acute pancreatitis development, ROS responsive PAMAM might have promising therapeutic implication in clinic.

#### 4.1.2. Antioxidative metal nanomaterial

Ce_2_O_3_ NPs and Y_2_O_3_ NPs have been reported to reduce the severity of pancreatitis injury after diazinon exposure (Khaksar et al., 2017). Nanoceria is a unique nanomaterial due to its peculiar redox regulation capabilities as compared with various other common metal nanoparticles including Se, Ag and Fe. Nanoceria can interchange between Ce^3+^ and Ce^4+^ oxidation states thus exert profound free radical scavenging activity. Given its catalase and SOD mimetic property, scientists probed the anti-pancreatitis activity of nanoceria against a cerulein-induced murine model of acute pancreatitis by multiple biochemical studies (Khurana et al., 2019). Nanoceria was found to be effectively internalized into macrophages and alleviated the oxidative and nitrosative stress induced by LPS. Analogously, yttrium, as an antioxidant with lanthanide-like electron configuration, has also been applied as Y_2_O_3_ nanoparticles in acute pancreatitis caused by cerulein hyperstimulation (Khurana et al., 2019). With a smaller size range of 1–100 nm, Y2O3 nanoparticles could selectively accumulate at the inflammatory pancreas via the ELVIS effect, and effectively exhibit remarkable protection against LPS-induced ROS production, MMP alterations and superoxide radical generation. More important, Y_2_O_3_ nanoparticles showed no significant cytotoxicity on macrophage up to a concentration of 0.1 mg/mL, indicating the high biocompatibility as a metal nanoparticle. The therapeutic mechanisms of Y_2_O_3_ nanoparticles involve the reduction of mitochondrial and ER stress by attenuating the Nrf2/NF-κB pathway. However, as for the metal nanoparticles, it still needs more experimental data to confirm the in vivo fate after administration and also the potential toxicity on the healthy tissues.

#### 4.1.3. Propanediamine derived delivery system

Studies have confirmed that propanediamine derivatives can penetrate through the BPB. It has been reported that Idoine-123 labeled N, N, N′-trimethyl-N′-(2-hydroxy-3-methyl-5-iodobenzyl)-1, 3- propanediamine (HIPDM) showed high accumulation in the pancreas for imaging in vivo (Yamamoto et al., 1985; Saji et al., 1991). Researchers suggested that HIPDM could be selectively accumulated at pancreas by the blood flow which governed by the pH gradient and then detained in the pancreas due to the ionic binding (Saji et al., 1991). One important bioactive compound that derived from traditional Chinese medicine, Rhein, was reported to treat acute pancreatitis using HIPDM modification strategy (Li et al., 2015). Rheinic acid (4, 5-dihydroxyanthraquinone-2-carboxylic acid) is an effective anti-inflammatory agent that is ineffective against acute pancreatitis due to its lack of specific accumulation in the pancreas (Cai et al., 2012). Li et al. selected the most suitable ligand, N,N,N′-trimethyl-benzyl)-1,3-propanediamine (HPDM) for Rhein, designed the HPDM-Rhein conjugates through formation of phenyl ester bonds and achieved optimal plasma stability, effective pancreas targetability, and remarkably improved cell delivery efficiency in the pancreas via the organic cation transporters. As compared to native Rhein, HPDM-Rhein significantly attenuated pancreatitis damage characterized by decreasing inflammatory cell infiltration, acinar cell vacuolization and acinar cell necrosis. Another research have demonstrated that a kind of complexes of 1-[2-(acridin-9-ylamino)ethyl]-1,3-dimethylthiourea and propanediamine can be embedded in into liposomes and is promising for non-small cell lung cancer (NSCLC) therapy (Zhou et al., 2019). These evidences demonstrated that propanediamine derivatives could serve as a good candidate for the development of multi-targeted ligands for treating pancreatitis. The propanediamine modified prodrugs also have the potential to be constructed into the nanoparticulate structure, which also contributed to the prolonged circulation, selective pancreas distribution, and improved therapeutics. From this point, the propanediamine based delivery strategies represents an efficient and safe strategy for acute pancreatitis management.

**Figure 5.**
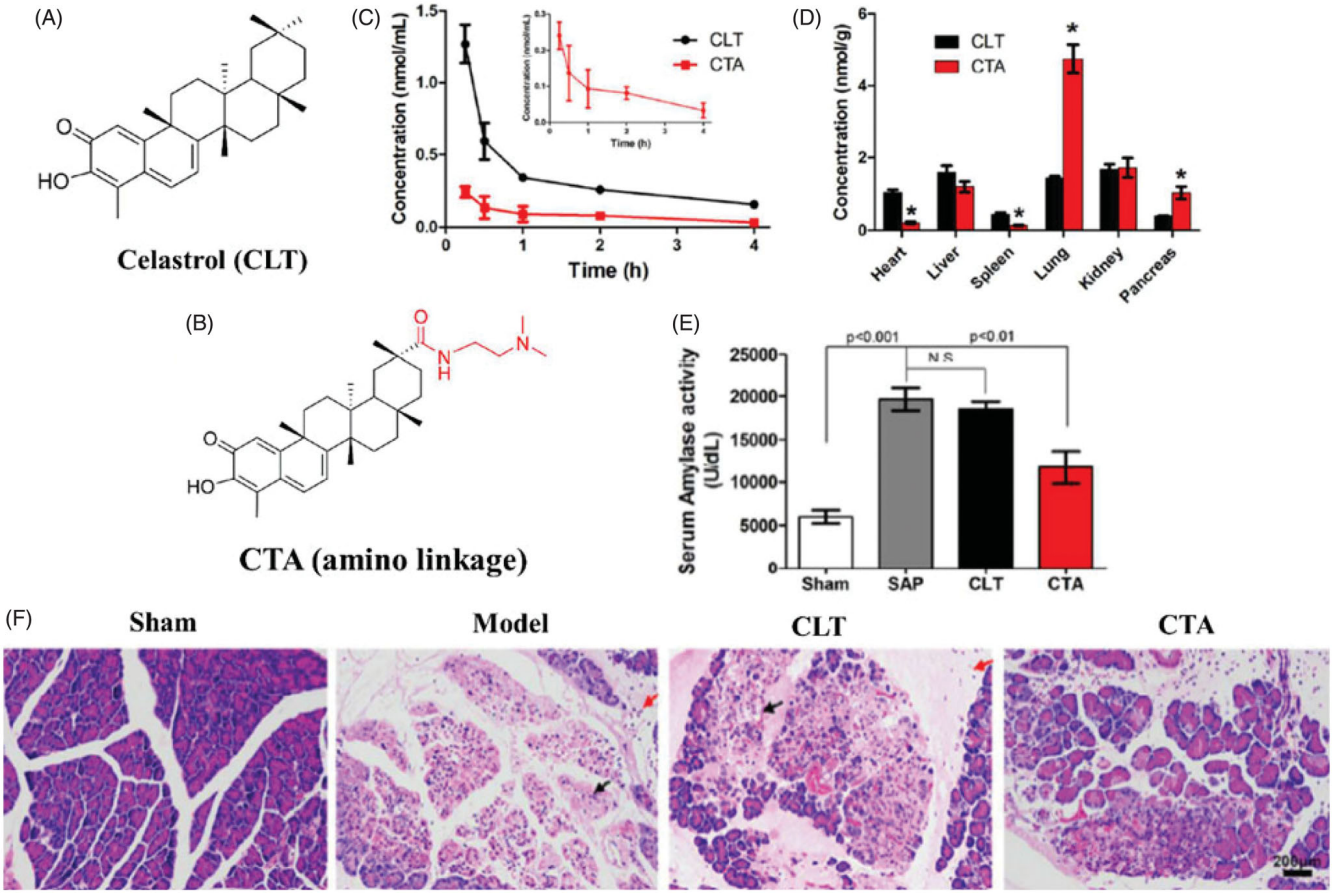
(A) Schematic diagram of structure and therapeutic outcomes of propanediamine inspired celastrol prodrug (CTA). (A) Chemical structure of celastrol (CLT). (B) Chemical structure of CTA. (C) The mean plasma concentration-time profiles of CTA and CLT in rats after intravenous injection. (D) Tissue distribution of CTA and CLT in rats 15 min after intravenous injection. (E) The serum amylase in pancreas following CTA treatment. (F) Representative H&E staining images of pancreas tissue following CTA treatment (Luo et al., 2017).

Inspired by the selective biodistribution of propanediamine derivatives, Luo et al. designed a the tertiary amine residue based prodrug for acute pancreatitis treatment (Luo et al., 2017). As illustrated in Figure 5(A,B), N, N-dimethyl tertiary amino group, was covalently conjugated to celastrol (CLT) to afford tertiary amino conjugates via either an ester (CP) or an amide linkage (CTA). CLT is a pentacyclic triterpenoid extracted from traditional Chinese medicine Tripterygium wilfordii, and has been reported to exert effective therapeutics effects in various inflammatory disease. In vitro study indicated that the CTA significantly increased the plasma stability and enhance the organic cation transporters mediated cellular uptake in acinar cells. In vivo distribution study confirmed that CTA could prolonged the circulation time and specifically distribute to the pancreas in rats, resulting in a 2.5-fold increase in the pancreas as compared to CLT (Figure 5(C,D)). After intravenous administration of CTA, tissue pathological score was greatly lowered, and the levels of pro-inflammatory cytokines were significantly downregulated in sodium taurocholate-induced acute pancreatitis rats (Figure 5(E,F)).

#### 4.1.4. Lipid and polymeric nanoparticles

The application of the lipid and polymeric nanoparticles was widely studied as drug delivery carriers in various disease. Recent studies indicated that these lipid and polymeric nanoparticles could also be applied to pancreatitis therapy. Somatostatin (SST) is a peptide hormone that regulated the endocrine system via different somatostatin receptor isoforms. SST has great potential in the treatment of acute pancreatitis, but its clinical application is hampered by the short half-life in the human body, which is only a few minutes. Cervin et al. designed a lipid-based liquid crystalline nanoparticulate as a carrier to delivery SST, which significantly increased the biological half-life of SST from a few minutes to longer than one hour (Cervin et al., 2009). Compared to the naturally derived lipid nanoparticles, polymeric nanoparticles could also be made from synthetic polymers. Poly(lactic-co-glycolic acid) (PLGA) nanoparticles often serve as a biodegradable and biocompatible platform for drug delivery. Yang et al. constructed PLGA nanoparticles to delivery genes for both tumor and acute pancreatitis targeted delivery (Yang et al., 2015). In this study, chloroquine diphosphate (CQ) compacted pDNA was loaded into the hydrophobic core and obtained the final nanoparticles (CQ/pDNA/PLGA NPs). The CQ/pDNA/PLGA NPs not only increased the transfection efficiency that resulted from the CQ codelivery but also increases the tumor targeting efficiency as well as circulation time in CT26-bearing mouse model. More importantly, these CQ/pDNA/PLGA NPs also showed high targeting efficiency in C57 acute pancreatitis model. This study is the first report on the targeted drug delivery of PLGA nanoparticles to pancreatitis animal model. Two reasons might contribute to the selective distribution of PLGA nanoparticles in the inflammatory pancreas: (1) PLGA nanoparticles could be uptake by the macrophage and neutrophils (Qiu et al., 2013), which were abundant in the inflammatory pancreas; (2) PLGA nanoparticles could selectively accumulate at the inflammatory lesions by the previous introduced ELVIS effect through leaky vasculatures. The novel PLGA-based gene delivery system showed high targeting efficiency for the inflammatory pancreas and demonstrated the therapeutic potential of PLGA-based nanoparticles in acute pancreatitis.

#### 4.1.5. Biomimetic nanoparticles

Many efforts have been taken to the embellishment of the nanoparticulate structure to evade systemic clearance from the reticuloendothelial system and selectively travels to the inflamed lesions. One recent emerging strategy is fabricating inflammatory cell mimic nanoparticles as drug delivery to achieve specific inflammatory microenvironment targeting. Among these, cell membrane coated nanotherapeutics has achieved satisfactory targeting efficiency in the inflamed tissue by retaining the specific receptor on the cell membrane (Yan et al., 2019). For the inflamed lesion targeting, the cell membrane usually extracted from the inflammatory cells, such as neutrophils, and macrophages.

**Figure 6.**
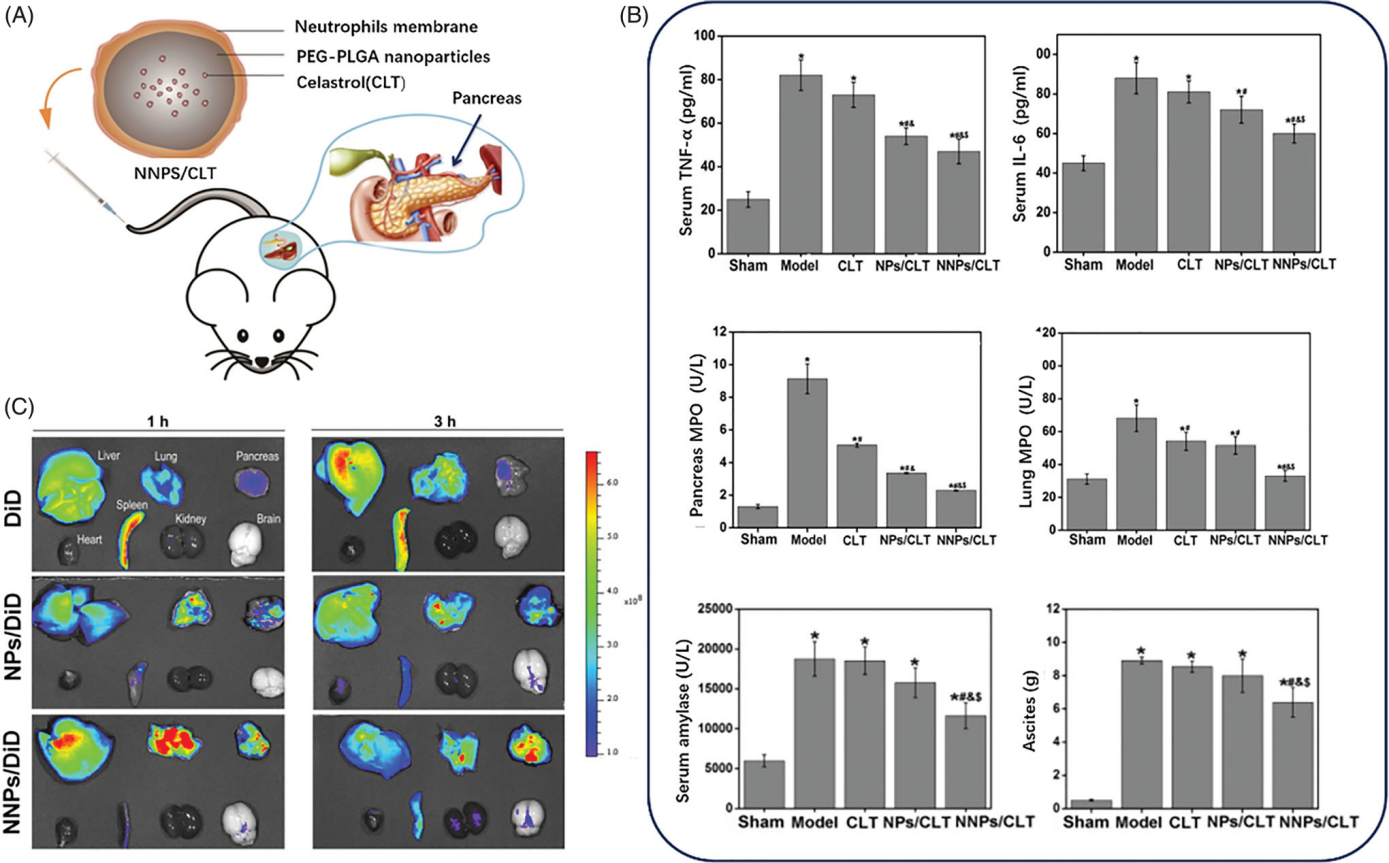
(A) Schematic diagram of structure and administration of CLT loaded PEG-PLGA nanoparticles coated by neutrophils membrane (NNPS/CLT) for acute pancreatitis therapy. (B) Pro-inflammatory cytokines level, and MPO level, serum amylase level and ascites in each group treated by CLT or CLT loaded PEG-PLGA nanoparticles (NPS/CLT) or NNPS/CLT. (C) Drug distribution in each group treated by DID labeled NPS (NPS/DID), DID labeled NNPS (NNPS/DID) was investigated by imaging analysis (Zhou et al., 2019).

The neutrophils usually act as a pioneering role in the inflammatory infiltration process during acute pancreatitis development. Zhou et al. developed a biomimetic nanoparticle (Figure 6) by neutrophils membrane coating method to delivery celastrol for acute pancreatitis (Zhou et al., 2019). Celastrol was validated to inhibit the NF-κB activation by targeting the Cys-179 in the activation loop of IKKβ to exert the anti-inflammatory effect (Lee et al., 2006), which could be applied as a potential anti-inflammatory agent in various inflammatory disorders (Kannaiyan et al., 2011). Celastrol biomimetic nanoparticles that coated with inflammatory cell membrane achieved pancreas targeting given the quickly and sensitively neutrophils infiltration to the injured acinar cells in acute pancreas (Zhou et al., 2019). Additionally, normal nanoparticles could check the innate system and increased the accumulation in the inflamed pancreas through the leaky vascular endothelium via ELVIS effects. As expected, the neutrophils membrane assisted nanoparticles in accumulating at the inflamed pancreas by the recruitment of inflammatory cells.

Carbon monoxide (CO) has anti-inflammatory effects involving the mitogen-activated protein kinases pathway. CO has been proven to be beneficial in the rodent model for the inflammatory bowel diseases (Takagi et al., 2015), sepsis (Liu et al., 2019), and ischemia-reperfusion (Ozaki et al., 2012), partially by inhibiting systemic inflammation. Studies indicated that exogenous CO release by CO-releasing molecular 2 (CORM-2) could be applied to reduce acute pancreatitis progression via inhibiting CD11bLy-6C monocyte migration and blocking CCR2 endocytosis (Wu et al., 2018). Inhaled CO has also proven to be quite tolerable in patients at a low dosage (Rosas et al., 2018). But several clinical trials using inhaled CO showed the difficulties in the dosage control and individual variability might limit its application. Since CO has a greater affinity for hemoglobin, hemoglobin has also been used to delivery CO, which has been applied as a biometric system in clinic (Taguchi et al., 2018). In addition to inhibiting acute pancreatitis, CO-hemoglobin also reduces the subsequent acute lung injury associated with pancreatitis. It may be due to the inhibition of systemic inflammation, lung neutrophil infiltration and HMGB-1 production. Biomimetic CO delivery based on the hemoglobin vesicle was also found to improve acute pancreatitis treatment by regulating the polarization of its macrophages toward M2-type (Taguchi et al., 2018). Peritoneal puncture and drainage can improve the therapeutic effect of acute pancreatitis by regulating the M2-type polarization of macrophages (Liu et al., 2018), which suggests that macrophages might serve as therapeutic target in pancreatitis.

#### 4.1.6. Stimuli-responsive nanoparticles

By observing the diverse environmental factors in the inflammatory sites, such as acidity and elevated enzyme, a few responsive drug delivery systems have been constructed to increase the efficacy and decrease the side effects through elevating the drug distribution in the inflamed site (Yao, Chen, et al., 2017; Yao, Huang, et al., 2017; Kou et al., 2020; Yao et al., 2020). Responsive nanoparticles have shown significant potential for delivering antibiotics to the site of infection, which is beneficial to relieve necrosis in acute pancreatitis. A pH-responsive lipid-dendrimer hybrid nanoparticle (LDH-NPs) was designed for Vancomycin (VCM) delivery to the site of bacterial infection (Amiti et al., 2019). PH-sensitive lipids (PSLs) with three hydrocarbon tails and a head group with a secondary amine and carboxylate function has formed liposomes to deliver VCM leading to better antibiotic therapy at acidic pH (Zhao et al., 2018). The pH-responsive nanoparticles have been applied in cancer therapy (Lamberti et al., 2015), as well as many other inflammatory diseases (Yang et al., 2018).

**Figure 7.**
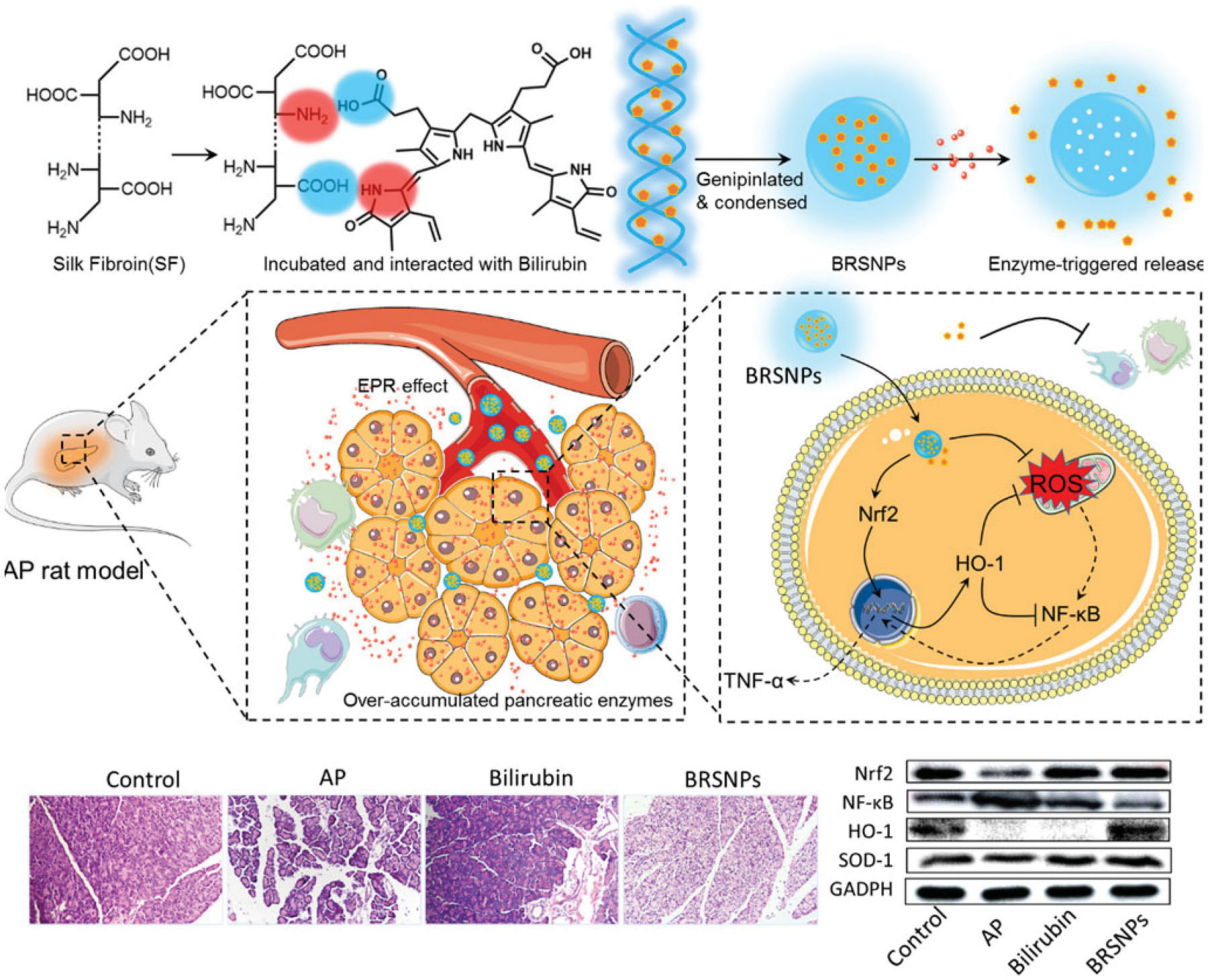
Schematic graph of bilirubin loaded silk fibroin nanoparticles (BRSNPs) for the experimental acute pancreatitis application. The developed BRSNPs could selectively accumulate at the inflamed pancreas and release the bilirubin in a trypsin-responsive manner. The mechanisms of BRSNPs against acute pancreatitis involves the inhibition of NF-κB signaling and activation of Nrf2/HO-1 signaling (Wang et al., 2016).

As mentioned above, the over premature release trypsin is one of the pathological features in acute pancreatitis. The over accumulated enzymes act as a stimulus for the nanoparticulate delivery system and could be utilized for controlled drug release (Yao et al., 2018; Qiao et al., 2019; Yao et al., 2019). Scientists have designed novel specific lipase-responsive nanoparticles as pancreatitis diagnostic agent (HW et al., 2014). We developed a trypsin responsive bilirubin nanoparticles using silk fibroin as matrix (BRSNPs) for the treatment of acute pancreatitis (Figure 7) (Wang et al., 2016). The constructed BRSNPs could selectively accumulated at the inflamed pancreas by the ELVIS effect and ROS responsiveness. And then the encapsulated bilirubin could be quickly released from the nanoparticles in the inflammatory pancreas that rich in the over-accumulated trypsin. Therefore, as expected, BRSNPs exerted strong therapeutic effect against acute pancreatitis by alleviating the inflammation in both pancreas and other organs, which involved the modulation of NF-κB and Nrf2/HO-1 pathway. As compared to the free drug injection, BRSNPs showed significantly enhanced delivery efficacy and promptly drug release of bilirubin, and resulted in better therapeutic effect. Therefore, stimuli-responsive nanoparticles that targets the microenvironment of inflamed pancreas could concentrate the drug in the pancreatic lesions and provided a viable delivery option for therapeutics against acute pancreatitis.

### 4.2. Other formulations for acute pancreatitis

#### 4.2.1. Chemical modification of the peptide-based therapeutics

Many pancreatic secretory trypsin inhibitors are peptides, which usually suffered from fast clearance after intravenous injection. Chemical modification of the peptides could optimize the circulation time by adjusting the peptide structure. For example, octreotide (Shandeshi Company), synthetic octapeptide complex, is the analogue of somatostatin. By removing the enzyme degradation point, octreotide significantly increased the enzyme resistance as compared to that of somatostatin and elevated the half-life of subcutaneous administration to around 100 min (Chanson et al., 1993).

#### 4.2.2. Injectable thermosensitive gel

The injectable thermosensitive gel provides a prolonged release after intramuscular or subcutaneous injection. Gabexate mesylate (GM) was developed as an example of the injectable thermosensitive gel (GMT1) by intramuscular injection (Gao et al., 2015). GM is a non-peptide trypsin inhibitor and often quickly removed by the body, making it difficult to manage and control the pharmacokinetic characteristics, especially when the blood supply of acute pancreatitis is often checked. Thus, GM is often clinically administrated by intravenous infusion to maintain the constant and effective blood drug concentration. By delivering with an injectable thermosensitive system, GMT1 could not only improve the pharmacokinetics characteristics but also successfully reduce the severity of traumatic pancreatitis. With the adjustment in the administration route, the injectable thermosensitive gel provides an alternative for the current delivery of trypsin inhibitors, which is of clinic importance.

#### 4.2.3. Emulsions

Emulsion preparation has been used to improve the distribution of drugs in acute pancreatitis. Aprotinin is a monomeric globular polypeptide and function as an extensive inhibitory action with particular activity against trypsin, chymotrypsin and kallikrein in the acute pancreatitis development (Smith et al., 2010). However, a recent meta-analysis confirmed that there is no solid evidence that supports the intravenous use of protease inhibitors could decrease the death rate in patients with acute pancreatitis (Seta et al., 2004, 2014). Its application is limited owing to its poor targetability at the pancreas, and it might trigger renal dysfunction when used combined with angiotensin-converting enzyme (ACE) inhibitors (Fritz & Wunderer, 1983; Waxler & Rabito, 2003; W UK, 2005). 99mTc-aprotinin loaded microemulsion (99mTc-aprotinin-M) was designed to increase the targetability for acute pancreatitis treatment (Ilem-Ozdemir et al., 2016). The 99mTc-aprotinin solution was also intravenously injected for comparison. While the highest concentration of aprotinin was found in the kidney for both formulations, much higher 99 m TC-aprotinin was detected in the blood in the 99 m TC-aprotinin emulsion group. This microemulsion formulation widens the application of aprotinin by changing the distribution of aprotinin after intravenous injection. Moreover, 99 m TC-aprotinin emulsions suppressed the median histopathological score and attenuated pancreatitis (Karasulu et al., 2015). The emulsion was recognized as a promising formulation for aprotinin in the acute pancreatitis treatment.

### 4.3. In vivo diagnostic imaging for detection of acute pancreatitis

Emerging nanoparticulate approaches also provide a new alternative for the diagnostic imaging of acute pancreatitis. Traditional diagnosis of pancreatitis usually restricted to the analysis of serum amylase and lipase. The in vivo diagnostic imaging could provide more information on the progression of acute pancreatitis in the clinical diagnosis. The most common nanoparticulate approach is the superparamagnetic iron oxide nanoparticles (SPIONs) before imaging the iron particles with an MRI scanner. Intravenously administrated SPIONs can be specifically ingested by macrophages reticuloendothelial system. Since intrinsically magnetic material of SPIONs and vast pro-inflammatory cytokines production induced by macrophages in pancreatitis, SPIONs was used as macrophages labeling agent in preclinically and clinically by MRI (Zhang et al., 2010). MRI imaging of SPIONs labeled macrophages homing to the kidney may facilitate early detection of the pathogenesis of kidney injury in acute pancreatitis, which contributes to symptomatic treatment as soon as possible. SPIONs also could be utilized for theranostic application by conjugating to the drugs. Clodronate is a synthetic bisphosphonate with poor cellular membrane permeability and a short half-life in the systemic circulation, which is often used for treating bone changes caused by osteoporosis (Han et al., 2019). Dang et al. developed a SPIONs loaded liposomes for macrophage labeling and MRI imaging, while the loaded clodronate could exert therapeutic activities against SAP (Dang et al., 2014).

Another approach is to use a metal chelator (e.g. diethylenetriaminepentaacetic, DTPA) which coordinate a radiotracer (68Ga, 111In, 64Cu) which can be imaged by PET or SPECT. The previous study has synthesized a Gadolinium diethylenetriaminepentaacetic fatty acid (Gd-DTPA-FA) nanoparticles as a diagnostic tool for the early identification of acute pancreatitis (HW et al., 2014). Gd-DTPA-FA has significantly increased the intensity of the T1-weighted MRI signal from 1 h to 36 h in an L-arginine-induced acute pancreatitis rat models (HW et al., 2014). Encouraged by this idea, a novel Gd-DTPA loaded liposomes (named M-Gd-NL) exhibited the ability to discriminate mild pancreatitis and severe pancreatitis as evidenced by a higher T magnetic resonance imaging signal in SAP than in MAP (Tian et al., 2017). These studies constituted a proof-of-concept for the utilization of nanoparticles in pancreatitis imaging and diagnosis, which is still challenging to move forward into clinic translation.

## 5. Conclusions and future directions

Acute pancreatitis is an inflammatory disorder that can be life-threatening in severe cases. For decades, the exact pathophysiological mechanisms of acute pancreatitis remained an enigma which was broken along with the emergence of the concept of autodigestive disease. At present, it was confirmed that the pathogenesis mainly involves premature trypsinogen activation, pathological calcium overloading, pancreatic microcirculation disorders, activation of NF-κB pathway, infiltration of leucocyte cells, and impaired autophagy. More depth mechanisms study still needed to explore more potential targets for acute pancreatitis.

Although timely clinical management such as fasting, nutritional support, fluid therapy to improve treatments for patients with acute pancreatitis are productive, there is still no unified criteria for fasting and nutrition. Drug therapy in reducing the secretion of gastric acid, inhibiting trypsin activity and secretion have been studied. However, the combined application of drugs is still in the sporadic observation stage. Traditional Chinese medicine has accumulated rich experience in the diagnosis and treatment of the digestive system. There is no unified standard for its clinical application, and its clinical operability is not strong.

Conventional formulations are used to improve the bioavailability, such as chemical modification. Novel drug delivery system provided new sight for treatment and diagnosis for acute pancreatitis, which is mostly devoted to the target delivery for increasing local drug accumulation. Lipid and polymeric nanoparticles are the most common strategies and platforms, which are biocompatible, biodegradable, and relatively stable in biologically relevant media, as well as incline to accumulate in the inflammatory sites owing to appropriate particle sizes and ELVIS effect. Biomaterials with therapeutic effect, such as PAMAM and metal nanoparticles, have also been explored to be applied in the treatment of acute pancreatitis and other inflammatory diseases. By cell membrane surface modification, bioactive drugs can be better transported to pancreas in acute pancreatitis by taking advantages of leukocyte infiltration. Further exploration can focus on the biomimic modification of biodegradable polymers and bioactive particles.

Although there are emerging batches of bioactive drug and novel drug delivery system for pancreatitis treatment, many of them are only used in a simple animal model which is insufficient for preclinical studies. The large-sample, multi-center study has not been carried out. Challenging large-scale trials, mechanistic studies and statist support are also needed to help us achieve more effective treatment in acute pancreatitis.
